# Dentistry responding in domestic violence and abuse (DRiDVA) feasibility study: a qualitative evaluation of the implementation experiences of dental professionals

**DOI:** 10.1186/s12903-023-03059-y

**Published:** 2023-07-12

**Authors:** Omolade Femi-Ajao, Janine Doughty, Maggie A. Evans, Medina Johnson, Annie Howell, Peter G. Robinson, Christopher J. Armitage, Gene Feder, Paul Coulthard

**Affiliations:** 1 The University of Manchester, Manchester, M13 9PL UK; 2 School of Dentistry, University of Liverpool, Liverpool, L69 3BX UK; 3 University of Bristol, Bristol, BS8 2PS UK; 4 IRISi, Bristol, BS8 2PS UK; 5 Queen Mary University of London, London, E1 2AD UK

**Keywords:** Domestic abuse, Domestic violence, Dentistry, Public health, Dental setting, Qualitative methods, Dental team

## Abstract

**Background:**

Domestic Violence and Abuse (DVA) is a persistent public health problem in the UK. Healthcare settings offer an opportunity to ask patients about DVA, either opportunistically or in response to the presence of injuries. However, it has been suggested that dental practices and dental teams have not been actively involved supporting adult patients when presenting with injuries that might have resulted from DVA. This qualitative study was conducted to satisfy the evaluative component of the Dentistry Responding in Domestic Violence and Abuse (DRiDVA) feasibility study.

**Methods:**

In total, 30 participants took part in the study; nine associate dentists and practice principals/owners took part in one-to-one interviews and 21 auxiliary staff took part across two focus group discussion sessions. Data were analysed using the seven step Framework Analysis process.

**Result:**

Three key themes were identified from the data, focusing on barriers to enquiring about domestic violence and abuse, Facilitators of identification and referral of DVA in dental settings, and recommendations for further adaptation of intervention to dental settings.

**Conclusion:**

DVA training coupled with robust referral pathways to a named specialist DVA advocate increases knowledge and awareness of the signs of DVA and confidence in making onward referrals. Further research is needed to understand how to increase dental professional willingness to ask patients about DVA.

## Introduction

Domestic Violence and Abuse (DVA) is a persistent public health problem in the UK. Annually, approximately 1.6 million women in England and Wales experience domestic abuse and almost one in three women (aged 16–59) will experience DVA in their lifetime [1, 2]. In England and Wales, two women are killed each week by current or previous abusive male partners [3]. DVA has negative physical and psychological impacts for individuals. Physical symptoms are commonly related to the nature of DVA, presenting as gynaecological issues, soft tissue bruising or bony fractures [4]. Psychological manifestations can include depression or post-traumatic stress disorder [5]. Healthcare settings offer an opportunity to ask patients about DVA, either opportunistically or in response to the presence of injuries. To date, UK-based interventions identifying and responding to DVA in healthcare settings have primarily focused efforts in accident and emergency departments, fracture clinics, primary medical care, and gynaecological and antenatal services [6, 7].

Facial injuries are reported in up to 75% of DVA cases; thus, the dental setting may present an untapped opportunity to ask patients about and identify DVA. Further, dental settings offer a discrete environment and long-standing relationships between dental professionals and patients may support disclosure [8]. Women affected by domestic abuse have expressed support for being asked about DVA by the dental team [9, 10]. However, dental professionals may feel that they lack the competence and confidence to identify DVA or to refer patients to appropriate DVA services [11, 12]. Existing evidence suggests that dental professionals would welcome more opportunities for DVA training, as such training is likely to enhance knowledge and competency to screen for and refer patients experiencing DVA [13, 14].

This qualitative study was conducted to satisfy the evaluative component of the Dentistry Responding in Domestic Violence and Abuse (DRiDVA) feasibility study [15]. The aim of the qualitative evaluation was to understand dental professionals’ experiences of participating in the study DVA training programme and delivering the intervention to the dental patient population.

## Methods

### The IRIS intervention (DVA training and care pathway)

The intervention used in the DRiDVA feasibility study was based upon the approach used by the IRIS (Identification and Referral to Improve Safety) specialist domestic violence and abuse (DVA) training, support, and referral programme for General Medical Practices. The IRIS DVA training has been comprehensively evaluated in primary care and has demonstrated intervention rate ratios of referrals to domestic violence services of 22·1 [95% CI 11·5–42·4]) and disclosures of domestic violence intervention rate ratio 3·1 [95% CI 2·2–4·3) when compared to control practices [16–18]. The intervention involves implementing the different components of the IRIS care pathway by training staff in general dental practice, providing a link to a designated advocate-educator from the collaborating agency, Manchester Womens’ Aid IRIS Team, and installation of software to prompt enquiring and recording of DVA information.

### Adapting the intervention to general dental practice

The IRIS programme described above was adapted to the dental setting with the intention of increasing the identification and referral of DVA among the dental patient population presenting with orofacial injuries. The intervention trained staff in general dental practices to identify risk factors for DVA, understand the role of the dental team, recognise signs of DVA in patients, ask patients about domestic abuse and support disclosure and referral to a specialist advocate. Additionally, the intervention provided a link to a designated advocate-educator from the collaborating agency, Manchester Womens’ Aid IRIS Team. Alongside the study researchers a Patient and Public Involvement (PPI) group, including victims and survivors of DVA, IRISi, Manchester Women’s Aid, Trafford Domestic Abuse Service, and Mankind, as well as dentists, dental care professionals and practice administrative staff adapted the training programme for use in the dental setting context. Dental-specific content included highlighting the importance and relevance of responding to domestic violence in dentistry including risk indicators e.g., facial injury. Further, training included a focus on DVA identification and response challenges specific to the setting.

### The DRiDVA feasibility study

The DRiDVA feasibility study aimed to measure the effectiveness of the adapted IRIS intervention using a cluster randomised trial design. There were two key sets of research questions and objectives:

The DRiDVA study aimed to answer two research questions:

1. Is it feasible and practical to use the IRIS care pathway (the intervention) within primary care dental practices?

The objectives used to understand care pathway feasibility included assessing the level of engagement of dental staff with the intervention training and support, and examining the acceptability of the intervention within primary care dental practices with a nested qualitative study.

2. Is it feasible to test the effectiveness of the intervention using a cluster-randomised trial design?

Three practices were randomly allocated to the intervention arm, and three to the usual practice (no intervention) arm. The primary outcome measure of intervention effectiveness was the number of referrals to a DVA advocate in the nine months following training of intervention practices. The secondary outcome measure was the DVA disclosure rate during this same period.

The objectives included measuring recruitment rates, examining reasons for attrition/non-participation, establishing outcome measures suitability, testing the feasibility of data collection for a trial in primary care dental practices, articulating health economics costs to inform the cost-effectiveness analysis in a definitive trial and estimating the sample size for an adequately powered definitive trial.

Key outcomes of the qualitative evaluation were to provide detailed information on the feasibility study process, adherence to the intervention, interpretation of the feasibility study findings, barriers and facilitators of implementation, level of engagement, experiences of dental practice staff on supporting adult patients experiencing domestic violence and abuse including managing disclosures, help-seeking, safeguarding and referrals.

#### Participant population and recruitment (sampling technique) for the qualitative study

As the DRIDVA study was exploring the feasibility of a practice-based intervention, the inclusion criteria for participating in the interview, was to have completed the DRIDVA training, and based in one of three intervention practices in North West England. Hence, study participants included dental professional, such as, dental nurses, associate dentists, and dental practice owners/principals, as well as, auxiliary staff, such as receptionists.

#### Methodological approaches: theory and epistemology

This nested qualitative study used the underlying theoretical and epistemological approaches used in previous studies on the IRIS intervention [16–18]. Specifically, the tenets of the Normalisation Process Theory (NPT) were used to understand how the experiences of study participants could potentially influence their willingness to implement what was learnt during the DRIDVA training.

### Data collection

All interviews and focus groups were conducted face-to-face by the lead author (OF) and were undertaken in participants’ dental practices. The interviews and focus groups utilised an interview schedule/topic guide and prompts to focus the sessions and lasted between 60 and 90 min. The interviews and focus groups were recorded and transcribed verbatim. Associate dentists and practice principals/owners were primarily responsible for providing the intervention and are the gatekeepers to practice; therefore, in-depth interviews were used to elicit their experiences in detail. Conducting focus groups exclusively with dental nurses and receptionists removed the hierarchical relationship between dentist and dental nurse which could have been prohibitive of open dialogue.

### Data analysis

Data were analysed using the seven step Framework Analysis process [19]. Stages one (transcription), two (familiarisation) and three (coding) were undertaken by researcher OF. Stages four through seven included developing and applying the framework, charting the data into the framework matrix, and interpreting the data; these stages were undertaken by three researchers (OF, MAG, and JD). Microsoft Word and Excel software were used to manage the data and the analysis processes. The data were presented as overarching themes and subthemes.

### Researcher reflexivity

The researcher (OF) conducting the qualitative evaluation has extensive experience of DVA research with a focus on help-seeking behaviours for female victims of DVA, and had volunteered as a helpline worker for a DVA charity. Other characteristics that may have impacted on the researcher’s conduct and interpretation include being female and never personally having witnessed or experienced DVA.

## Results

In total, 30 participants took part in the study; nine associate dentists and practice principals/owners took part in one-to-one interviews and 21 auxiliary staff took part across two focus groups. The participants were predominantly dental nurses and associate dentists, most were female and very few had any experience of patients disclosing experiences of DVA (Table 1). Four themes were derived from the analysis of the interview and focus group data, these explored practical issues concerning the appropriateness of the intervention to the dental setting, and dental professionals’ experiences of training and intervention delivery, and managing disclosures and referral processes. The deductive themes were guided by the objectives of the evaluation (Table 2).

**Table 1:** Characteristics of study participants

Characteristics		Interview *n = 9*	Focus groups *n = 21*
Dental professional role	Principal dentist / practice owner	3	0
	Associate dentist	6	1
	Dental nurse	0	16
	Receptionist	0	4
Gender	Male	5	1
	Female	4	20
Time since qualification	0–5 years	5	-
	5–10 years	1	-
	10–15 years	2	-
	15 + years	1	-
Time at current practice	0–5	6	-
	5–10	2	-
	10–15	0	-
	15 +	1	-
Past experience of DVA disclosure	Never	6	19
	Single occasion	2	2
	Multiple occasions	1	0

**Table 2:** Study themes and subthemes

Themes		Subthemes
Theme 1	Barriers to identification and referral of DVA in dental settings	Encountering patients who were reluctant to disclose DVA Feeling uncertain about professional responsibilities and DVA Anticipating uncomfortable feelings when talking about DVA
Theme 2	Facilitators of identification and referral of DVA in dental settings	Undertaking training Local ownership of the intervention Long-standing relationships with patients Triggers to ask about DVA Experiencing a DVA disclosure Structured referral pathway and named DVA advocate
Theme 3	Recommendations for further adaptation of intervention to dental settings	N/A

## Theme 1: barriers to enquiring about DVA

The following section describes the barriers that limited the translation of training and referral into real-world behaviour change.

### Encountering patients who were reluctant to disclose DVA

Dental professionals understood reasons that patients may not disclose DVA, including feeling too scared or frightened, protecting the abuser or themselves, preventing embarrassment, or being mentally unprepared to disclose at that time. Dental professional participants appreciated the difficulty of disclosure and the big step it represented for victims. Clinicians were empathetic and sensitive toward patients who chose not to disclose. When patients opened up easily or straight away or there were clear physical manifestations, dental professionals felt more confident to ask about DVA. However, when DVA was suspected but the patient denied any problems, dental professionals found it intrusive to continue the line of questioning.

*“Well, it must have been immensely difficult, and you could tell with the agitation and the anxiety, just how–, you know, non-verbal communication, you could tell it was very difficult. She was shaking, you know, but I think that once she actually said it and the words left her lips, I think you could tell there was a bit of like–, bit of relief”* [Female dentist, 30 + years experience].

“*I actually felt more comfortable ‘cause she wanted to open up to me but if I had to probe here, I feel like it would have been different.”*

### Feeling uncertain about professional responsibilities and DVA

Some dental professionals questioned whether the intervention was aligned with their professional responsibilities and scope of practice. As a result, they were uncertain as to whether patients would consider DVA questions appropriate from a dental professional. Screening for DVA felt awkward for many dentists because they felt patients don’t expect to be asked about DVA during their dental appointment. Participants expressed mixed views about the appropriateness of identification and referral of DVA in dental settings. Most dental professionals believed that they were aptly placed to identify DVA, had a responsibility to act to support and protect patients, and thus considered the intervention appropriate to their role. However, this view was not universal. Some dental professionals recommended taking a less active approach to identifying and managing DVA and felt that their role should be limited to patient referral to DVA services. Taking part in the intervention improved participants perceptions of the appropriateness of the role of the dental professional in screening for DVA. One participant, a newly qualified male dentist, had encountered a patient who had previously been hospitalised for DVA and had “assumed that the hospital would have taken care of all that kind of stuff before she came to me” [Male, associate dentist, newly qualified].

### Anticipating uncomfortable feelings when talking about DVA

Translating improved knowledge and awareness to real-world behaviour change was stymied by the perceived risk of subsequently having to negotiate uncomfortable conversations with patients; this was a strongly felt sentiment that was mentioned by almost all participants, often multiple times. Though dental professionals felt more comfortable with negotiating referral pathways following training, many remained anxious about asking patients about DVA. A minority of dental professionals had not asked any patients about experiences of DVA. The topic of DVA and the stigma and shame associated with it made it a difficult subject to bring up and dental professionals believed that patients may wish to hide their experiences. Clinicians who failed to ask about DVA explained that having conversations about DVA was deeply uncomfortable for them. One clinician even believed that, following training, they had missed an opportunity to ask a patient with suspected DVA due to their acute discomfort with initiating the conversation. Contrastingly, others who had not asked the question still explained that following training, they felt empowered and confident to do so, but had not been triggered to do so during the study period. The more uncomfortable the clinician felt about asking about DVA, the less likely they were to be willing to ask the question. More recently qualified dentists tended more toward nervousness about initiating DVA conversations than experienced dentists, which may have resulted from their general lack of clinical experience. However, dental nurses were generally less anxious about asking about DVA and initiating referrals than the dentist.

*“How do I feel? Probably quite awkward about it, not confident” [Male, associate dentist, qualified less than 10 years].*

*“I don’t think with myself, necessarily, it’s changed how I would practice, but it’s highlighted things that I wouldn’t necessarily think of and who to refer to, rather than just going about the same pathway, but not really within my questioning.”* [Female, associate dentist, qualified less than 10 years].

## Theme 2: facilitators of implementing the intervention

### Undertaking the training session

Before the training, dental professionals explained that they lacked the confidence to approach the topic of DVA with their patients. They reflected that a lack of knowledge prior to training likely resulted in missed opportunities to identify DVA. For most, the training programme alleviated anxiety about managing DVA disclosure and referral processes. As a result, dental professionals’ confidence and willingness engage with the intervention delivery increased.

*“Prior to the training it would have been a difficult subject to bring up because if someone did then report that they were having issues we wouldn’t have really known how to immediately follow it up, but now we know what, exactly what action we would take, which makes you more confident in bringing the subject up.”* [Male practice owner, qualified nine years].

### Local ownership of the intervention

Local ownership and developing ways of team-working helped the intervention practices to ask patients about DVA identification. For example, teams explored ways to integrate dental nurses into intervention delivery to minimise the impact to dentists’ workload. Dental nurses described their role as advocates for patients, they strongly believed that the intervention offered benefits and were empowered by the training to act on their intuition:

*“I mean if I noticed something that a dentist didn’t, I would make it obvious that I’ve noticed this, have you noticed this? I wouldn’t just think, oh, the dentist hasn’t noticed it, I won’t say anything. I would speak up for the sake of the patient.”* [Female, dental nurse].

One experienced dentist thought that local ownership was supported by having more female staff and a younger team. However, these perceptions contrast with the findings of this study where less experienced dentists were less likely to ask about DVA despite their willingness to engage with the theoretical aspects of training.

Though some practices felt that finding a separate quiet and discrete space, that was comfortable and safe was important in the event of a disclosure, others believed that a separate room for disclosure added too much weight or seriousness to the encounter and preferred to have the conversation in their usual dental surgery environment. However, dental teams tended to consider the dental practice environment as an inherently safe space.

***“****I think being a dental practice owner we’re in an ideal position [to ask about DVA], especially when we get to know our patients as well we can. We’ll be able to respond to changes in behaviour and I think it’s [screening for and identifying DVA] of vital importance.”* [Practice owner and principal dentist, qualified more than 10 years].

### Long-standing relationships with patients

Several dental professionals felt that long-standing relationships with patients would encourage them to open up about DVA; this was related to building trust over time. One dental professional assumed that as the dentist-patient relationship developed, patients would be encouraged to discuss DVA. Contrastingly, another felt that long-standing relationships with patients and familiarity with their home situations amplified the discomfort with asking about DVA because it would “*stick out as being abnormal”* [Associate dentist, qualified 15 + years].

“*I think the more you get to know someone, then the more they’ll put up with you because as a stranger, you like to keep everything to yourself; whereas if you gain trust, especially in an intimate environment looking people’s mouths, they gain trust each time they meet you. I wouldn’t say it’s something that’s gained automatically within the first appointment. It’s something that you get over time.”* [Associate dentist].

### Triggers to ask about DVA

The DRiDVA intervention specifically pertained to asking patients with facial or dental injury about DVA. In some instances, dental professionals probed patients for further information when their explanation was not consistent with the presentation of the injuries. In other encounters patients actively volunteered information about their experiences of DVA. Another factor that prompted dental professionals to enquire included long-standing lack of dental attendance. Many dental professionals were surprised to learn the value of simply asking about DVA to encourage either immediate disclosure or to initiate a process of considering disclosure in the future: “*at least we’ve planted a seed”* [Practice owner, 10–15 years qualified].

*“I went to ask the patient about what happened and the story that was given and the injuries didn’t match. ....I could tell she was becoming quite agitated which just heightened my suspicion that something else was going on. So then, I just asked her the direct question of like, “Did someone do this to you?”* [Practice owner and principal dentist, qualified more than 10 years].

### Experiencing a DVA disclosure

The perceived value of the intervention was heightened when dental professionals encountered patients who disclosed experiences of DVA. Dental professionals who had experienced a disclosure believed that the intervention had the potential to benefit the patient population and was a worthwhile endeavour. Therefore, reinforcement of the benefits of the intervention was not experienced by those who did not ask the question. Participants believed that dental professionals may be encouraged to engage with the intervention if the experiences of these dental professionals were captured in a video format that could be delivered in training sessions.

*“It must have been immensely difficult, and you could tell with the agitation and the anxiety, (…) non-verbal communication, you could tell it was very difficult. She was shaking, you know, but I think that once she actually said it and the words left her lips, I think you could tell there was a bit of like, bit of relief* “[Practice principal, qualified more than 10 years].

### Structured referral pathway and named DVA advocate

During the 9-month delivery phase, all disclosures of DVA across the three intervention practices accepted referral to DVA services. Although the dental team described being uncertain what would happen once a referral was made, they were reassured when they received positive responses from patients about their experiences moving onward from the dental practice. Having a structured referral pathway and face-to-face contact with a named advocate educator gave dental professionals greater confidence to initiate referrals.

*“I think it makes it easier the first time we do it, but it’s the figure head role [advocate educator], it doesn’t really matter who it is, as long as you have somebody that, this is where we go to. I think it has made it easier because we’ve seen a face”* [Female, associate dentist, qualified more than 30 years].

“*I was with one of the dentists and I got the feeling that it was something I needed to do there and then so I asked the patient, “Would you like me to ring her now?” you know, after we’d gone through all the what we can do […] she actually spoke to [nominated DVA point of contact] that day and that started it all off, because I thought if she goes we might lose her, I thought we need to try and do it while she’s here*” [Female, head dental nurse].

## Theme 3: recommendations for further adaptation of the intervention to dental settings

Recommendations for future training and support included a marked preference for a single training session as opposed to delivery across multiple sessions. Some participants wished for written information summarising the training. Following training, a few participants felt that conversations with patients were still challenging and awkward; additional support in the form of scripted prompts, on-clinic observation, and feedback was recommended. Dental professionals requested the use of gender neutral (as opposed to female-orientated) terminology to refer to DVA victims during the training sessions; some participants were concerned that a gendered focus might result in missed opportunities to identify men experiencing DVA. There was overwhelming support for refresher courses, not only to update knowledge but also to ensure that screening for DVA remained at the forefront of the minds of dental team members. Refresher courses could also provide an opportunity for sharing experiences with, and learning from, others.

*“Although like I feel very confident now and it was sufficient, the amount of training was perfect, over the next like say year, two years, we could have like a refresher where we come back together and we either discuss people’s views or people’s referrals that they’ve done over the past year, and then people can learn from them referrals. So, it’s important to have some sort of follow-up”* [Dentist, qualified less than five years].

## Discussion

This study offers insight into real-world implementation of training dental professionals to implement DVA screening and identification. Further, it demonstrates the value of qualitative evaluation in eliciting the barriers and facilitators to introducing a novel complex intervention within the context of dental practice.

Despite the reticence to delivering the intervention voiced by some participants, most dental professionals were heartened by the positive experiences that occurred when they created space for patients to engage in conversations about DVA. Some patients were described as forthcoming about their DVA experiences even when the enquiry was relatively straightforward or unintrusive. For those dental professionals who had experienced a DVA disclosure, the value of the intervention was clear, and they were more likely to report positive thoughts about their role in delivering DVA screening and identification.

Before taking part in the DRiDVA training, knowledge deficits were identified by most dental professionals. Wider literature from Europe and the US has identified that insufficient DVA training is a universal issue within the dental profession [14, 20]. In the UK, authors have identified a paucity of undergraduate training in DVA, primarily due to full curriculums and lack of appropriately trained staff to deliver the training [21]. Many dental professionals who took part in the intervention reported that their confidence in asking about and referring patients experiencing DVA was enhanced through the knowledge and awareness-raising resulting from the training sessions. However, this did not necessarily translate to willingness to identify DVA among the dental patient population. This finding mirrors other studies reporting increases in dental professionals’ knowledge and self-efficacy following DVA training [22]. Other intervention studies undertaking training with dental students have found that despite greater knowledge, students still felt uncertain about identifying DVA and reporting cases [13]. Further, although knowledge improves with training, this does not necessarily translate into changes in attitudes and beliefs about DVA [23].

Following DRiDVA training, most dental professionals valued the potential for the intervention to benefit patients and highlighted how previously avoiding the topic of DVA may have led to missed opportunities for identification and referral. Other studies have found that following DVA training, dental professionals increasingly agree with the importance of DVA screening and identification as part of their role [24]. The dentists in this study feared that the unexpectedness of a dentist asking about DVA might be a trigger for patients to become offended or defensive. Fear of compromising the dentist-patient relationship is one of the most commonly encountered barriers across DVA studies with dental professionals [14, 25–27].

Maintaining good dentist-patient relationships and avoiding complaints are cornerstones of building a successful dental practice. Therefore, introducing a topic such as DVA, which may seem unexpected, confrontational or sensitive could be perceived as counter to the commercial aims of the business of dental practice [28, 29]. In this study, dental nurses were less anxious about asking about DVA when compared to dentists, this may have been because, as salaried employees, the patient-practitioner relationship was not directly linked to their ongoing source of remuneration. Further, DVA can provoke cultural, internalised and anticipated stigma which de-legitimise DVA experiences, perpetuate negative stereotypes about people who have experienced DVA and prevent help-seeking behaviours [30]. Healthcare professionals in other studies have reported that alongside practical challenges presented by the clinical environment and lack of support or training, societal beliefs that support victim blaming narratives create barriers by normalising DVA and undermining the credibility of victims who disclose DVA [31]. Nonetheless, broader literature has demonstrated that women who have previously experienced DVA consider DVA screening acceptable in the dental setting and wish to be routinely asked about their experiences of DVA [9].

Notable strengths of this study were that all clinical and support staff from dental practices delivering the intervention attended the training and the level of engagement and willingness to participate in research was high. However, despite the willingness of the staff at these dental sites to act as intervention champions in engaging with the intervention, there was still reticence to ask patients about DVA. Further thought is warranted about how to overcome barriers to asking about DVA as even willing and able dental professionals did not fully buy in to delivering the intervention. Additionally, there was no engagement from dental practices in the control arm, hence, the study was not able to evaluate how dental practices who had not received the study intervention would have engaged with patients disclosing DVA experience.

### Implications for practice

The perceived value of the intervention was improved when dentists encountered patients who disclosed experiences of DVA; other dental professionals may be encouraged to engage with the intervention if the experiences of these dental professionals was captured in a video format that could be delivered in training sessions. Additionally, dental teams recommended refresher meetings which could offer an opportunity to share experiences in identifying DVA and referring patients for support between dental practices. Other learning points included reducing the number of sessions taken to deliver the training and moving away from strongly gendered language around victims and perpetrators of DVA to avoid missing opportunities to encourage men to disclose DVA.

Further research may include understanding how to overcome dental professionals’ barriers toward discussing sensitive topics with dental patients, this may include role plays with DVA specialists or scripted encounters which offer an opportunity to practice negotiating conversations about DVA with challenging patients. Additionally, hearing from other dental professionals who have had positive experiences of asking the question or have identified someone affected by DVA may provide reassurance that talking about DVA is unlikely to compromise relationships with most patients.

## Conclusions

DVA training coupled with robust referral pathways to a named specialist DVA advocate increases knowledge and awareness of the signs of DVA and confidence in making onward referrals. However, training does not necessarily translate to increased willingness to routinely screen for DVA in dental practice. The initial training programme requires adaptation to more closely meet the needs of dental professionals working in primary dental care. Further research is needed to understand how to increase dental professional willingness to ask patients about DVA.

## Data Availability

The datasets generated and/or analysed during the current study are not publicly available due to ethical restrictions, but are available from the corresponding author on reasonable request.
